# The role of fossils for reconstructing the evolution of plant development

**DOI:** 10.1242/dev.204322

**Published:** 2024-10-17

**Authors:** Alexander J. Hetherington

**Affiliations:** Institute of Molecular Plant Sciences, School of Biological Sciences, University of Edinburgh, Max Born Crescent, Edinburgh, EH9 3BF, UK

**Keywords:** Plant evolution, Fossil plants, Root evolution, Shoot evolution, Meristem evolution

## Abstract

Many of the developmental innovations that underpin the diversity of plant form alive today, such as those facilitating apical growth, branching, leaves, roots, wood and seeds, all evolved over 360 million years ago. Fossils, as our only direct record of plant form in the past, are thus essential for interpreting the origin and evolution of these innovations. The focus of this Spotlight is to showcase the rich plant fossil record open for developmental interpretation and to cement the role that fossils play at a time when increases in genome sequencing and new model species make tackling major questions in the area of plant evolution and development tractable for the first time.

## Teaser

**Summary:** This Spotlight discusses how fossils, as our only direct evidence for plant form in the past, preserve essential lines of evidence for reconstructing the evolution of plant development.

## Introduction

A central goal of plant evolutionary developmental biology (evo-devo) is to characterise the developmental changes that underpinned the origin and diversification of land plant body plans. However, a major challenge in accomplishing this is that many of the characteristics of plant body plans evolved early. In fact, characteristics such as vasculature, branching, roots, leaves, and seeds containing bipolar embryos, had all evolved by the beginning of the Carboniferous Period roughly 350 million years ago (Ma) (Chomicki et al., 2017; Galtier and Holmes, 1982; Hetherington et al., 2020; Kenrick and Crane, 1997; Long, 1975). The ancient timing of these events makes piecing together the developmental changes that underpinned them challenging.

The most widely used approach in plant evo-devo to shed light on these events is to carry out comparative development studies of living species and use these analyses to make predictions about plant evolution in the past. This approach has been hugely informative and it is only through examination of living species that we can directly observe and experimentally manipulate the developmental processes of growth and differentiation in real time. Furthermore, because DNA has only been reported to survive in the fossil record for 1-2 million years (Kjær et al., 2022; van der Valk et al., 2021), comparative genomic analyses of living species offer our only opportunity to predict the genetic complement of extinct species in deep time (Bowles et al., 2020; Harris et al., 2022). However, a drawback of these studies is that living species alone can, in some cases, provide a poor lens for predicting evolutionary events in the past. This is because extinction may obscure patterns of character evolution through the loss of intermediate forms or loss of character combinations not observed in living species. Therefore, living species thought to preserve suites of ancestral characteristics may in fact be highly derived compared to their ancestors.

**Fig. 1.**
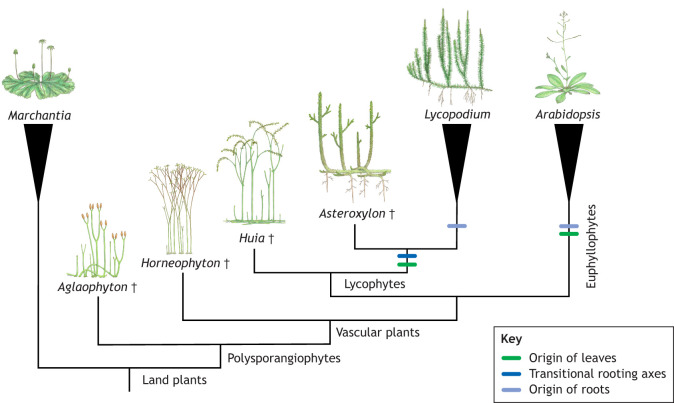
**Fossils indicate that roots and leaves evolved independently in vascular plants.** Schematic cladogram of living and fossil land plants based on Kenrick and Crane (1997). Full species names: *Marchantia polymorpha*, *Aglaophyton majus*, *Horneophyton lignieri*, *Huia gracilis*, *Lycopodium annotinum*, *Arabidopsis thaliana*. All extinct taxa are highlighted with the dagger symbol. Illustrations by Julianne Kiely.

Fossils, in contrast, provide our only direct evidence for plant form in the past and, therefore, hold an essential place for testing predictions about the evolution of development. Fossils have a long history in studies of evo-devo (Hall, 2012; Peterson et al., 2007; Raff, 2007; Shubin et al., 1997) and have been a growing force in the study of plant evo-devo over the last two decades (Ambrose and Ferrándiz, 2013; Boyce, 2005, 2008, 2010; Boyce and Knoll, 2002; Chomicki et al., 2017; Delaux et al., 2019; Dolan, 2009; Fernández-Mazuecos and Glover, 2017; Friedman et al., 2004; Harrison, 2017; Harrison and Morris, 2018; Hetherington and Dolan, 2017; Niklas and Kutschera, 2017; Petrone-Mendoza et al., 2023; Pires and Dolan, 2012; Plackett et al., 2015; Rothwell et al., 2008, 2014; Rudall and Bateman, 2019; Sanders et al., 2007, 2009, 2011; Strother, 2023; Tomescu and Groover, 2019; Tomescu and Matsunaga, 2019; Tomescu and Rothwell, 2022; Tomescu and Whitewoods, 2024; Vasco et al., 2013, 2016). Fossils have proved transformative for our understanding of plant evolution; for example, based on an investigation of living species alone, we might predict that roots and leaves only evolved once within vascular plants (Schneider et al., 2002). However, if we include fossils, then a very different picture emerges; the common ancestor of vascular plants is interpreted to lack leaves and roots and, instead, both evolved at least twice independently in vascular plants (Boyce, 2005, 2010; Friedman et al., 2004; Harrison and Morris, 2018; Hetherington and Dolan, 2018, 2019; Kenrick and Crane, 1997; Sanders et al., 2009; Tomescu, 2009; Tomescu and Whitewoods, 2024) (Fig. 1).

Fossils, therefore, become essential for how we interpret the development of living species. For instance, orthologous genes expressed in the roots or leaves of lycophytes and euphyllophytes (Harrison et al., 2005; Huang and Schiefelbein, 2015; Motte et al., 2022; Spencer et al., 2020; Yang et al., 2023), two major groups of living vascular plants in which leaves and roots evolved independently, cannot be predicted to have been expressed in comparable organs in a rootless or leafless ancestor. Although we cannot directly test patterns of gene expression or experimentally manipulate development within fossils they are ultimately as important as investigations of living species for building theories about the evolution of plant development. Here, I highlight how fossils, and especially fossils with exceptional preservation, are essential for studies of plant evo-devo owing to our ability to make inferences about development based on morphology and anatomy.

## How fossils can be used to make predictions about development in the past

### Inferring development from anatomy and morphology

Two connected features of plant development borne out by extensive studies of living species make it easy to integrate fossils into the study of plant evo-devo. First, owing to their relatively rigid cell walls, plant cells typically do not move during development (Steeves and Sussex, 1989). This means that by studying the cellular organisation of plants we can, in certain cases, make predictions about the two main developmental processes: growth (constituting cell division and enlargement) and differentiation. Second, development in plants continues post-embryonically as a result of the activity of meristems (Steeves and Sussex, 1989). Meristems are regions of plants that contain self-renewing populations of stem cells and their rapidly dividing daughter cells that give rise to the plant body. The two most well-studied meristems occupy the apices of shoots and roots and are termed the shoot and root apical meristems. Meristems iteratively, and often predictably, build up the plant body, meaning that the overall form of a plant preserves a record of its development. This can be observed at the micro-scale in the epidermis of roots or grass leaves. The epidermises of both are composed of linear files of cells formed by regular cell division, elongation and differentiation. Each file of cells preserves evidence of a developmental trajectory from small undifferentiated cells close to the meristem to differentiated stomata or root hairs (Dolan, 1996; Dolan and Costa, 2001; Hepworth et al., 2018; Raissig et al., 2017). At the macro-scale, a record of development is directly preserved in the arrangement of leaves, a characteristic termed phyllotaxis (Jean, 1994; Kuhlemeier, 2007), or even at the whole-plant scale, such as the modular habit of some trees that are built up of repeated units composed of branched shoots, leaves and buds (Hallé, 1986). These two features of plant development are important for evolutionary studies because if we can make predictions about development based on morphology and anatomy in living species, then we can do the same with fossils, a point well illustrated by the investigation of fossil wood.

### Wood evolution, a case study for the role of fossils in plant evo-devo

Wood is composed of secondary xylem that develops from the meristem termed the vascular cambium. During development, the cambium produces new secondary xylem inwardly that gradually increases the girth of the plant and displaces the vascular cambium further outwards (Creber, 1977). Wood preserves in its anatomy a record of growth and differentiation from the vascular cambium through time and the decay-resistant structure of wood means that it has a good chance of being preserved in the fossil record. The 400-million-year fossil record of wood (Strullu-Derrien et al., 2014) can be used for establishing when the cambium evolved and how it has changed through time. Today, a vascular cambium is only present in seed plants. However, as with the investigation of leaves and roots, fossils examined in a phylogenetic context indicate that the vascular cambium evolved multiple times during plant evolution (Boyce, 2010; Tomescu and Groover, 2019). The independent origin of a vascular cambium underpinned the separate origin of an arborescent growth habit in lycophytes, ferns and seed plants, shaping early forest ecosystems. Investigating the anatomy of wood in these extinct species also reveals that the cambium varied between different groups. For example, the now extinct tree lycophytes developed a vascular cambium that was almost solely unifacial, producing files of xylem inwards but producing no or almost no secondary phloem outwards, and fossils also preserve a large and now extinct diversity of different organisations (D'Antonio and Boyce, 2021; Cichan, 1985a,b; Tomescu and Groover, 2019). Studying the anatomy of fossil wood has even been proposed to preserve a direct record of polar auxin transport in extinct lineages of seed plants and lycophytes (Rothwell and Lev-Yadun, 2005; Rothwell et al., 2008; Sanders et al., 2011). Recognising the characteristic swirled anatomy of tracheids of living species today that results from polar auxin transport, palaeobotanists used this anatomical characteristic, which they term a ‘structural fingerprint’ of development, to predict the direction of auxin flow when comparable patterns were identified in fossils (Rothwell et al., 2014; Sanders et al., 2011; Tomescu and Matsunaga, 2019; Tomescu and Rothwell, 2022). Wood development continues (Pfeiler and Tomescu, 2023) to provide a great case study for how fossils can be integrated with investigations of living species to shape our understanding of the evolution of plant development.

### The importance of exceptionally preserved fossils

The lignified nature of wood means that it is more likely to be entombed in the fossil record compared with delicate meristems or fleeting stages in development, such as embryos. However, occasionally fossils with exceptional levels of preservation maintain these crucial development features. The majority of these fossils are preserved as permineralisations (Schopf, 1975; Stewart and Rothwell, 1993), which occurs when mineral-rich fluid permeates the plant tissue either while it is still alive or after death. These minerals, such as silica, calcium carbonate, iron sulphide and iron oxy-/hydroxides, are deposited within and around cells preserving their three-dimensional cellular anatomy (Channing and Edwards, 2009; Scott and Rex, 1985; Stewart and Rothwell, 1993). The process is analogous to the fixation and embedding steps carried out during classic histological investigation of living plant tissues. Thin preparations produced from permineralised fossils can be investigated side by side with histological preparations of living species. Sites of exceptional preservation occur in almost all geological periods covering land plant evolution (Channing et al., 2011; Edwards, 2004; Galtier, 1997; Shi et al., 2021; Slater et al., 2015; Stewart and Rothwell, 1993; Taylor et al., 2009) and frequently preserve cellular and even occasionally subcellular plant structure (Bomfleur et al., 2014; Gould, 1971; Niklas et al., 1978; Taylor and Millay, 1977). These fossils capture fleeting moments in plant development, such as the growth of pollen tubes (Rothwell, 1972), release of spermatozoids (Kerp, 2018) or the germination of spores (Scott, 1904). Cases such as these often provide the earliest evidence for developmental processes during plant evolution and, although the number of replicates for each is often minuscule (in many cases, a discovery may rely on a single fossil), their importance for piecing together plant evolution is invaluable. Exceptionally preserved fossils such as these can contribute to disparate aspects of the study of plant development but are of particular value for testing predictions about the origins of new organs and their distinct apical meristems in vascular plants.

## Fossil evidence for the evolution of apical meristems in vascular plants

Apical meristems have been centre stage in the study of plant development for the past 150 years. Early studies in the 19th century and the first half of the 20th century were largely focussed on comparative anatomical investigations (Clowes, 1961; Guttenberg, 1966; Hofmeister, 1862; Popham, 1951; Steeves, 2006; Van Tieghem and Douliot, 1888). Based on these investigations, it was recognised that apical meristems varied substantially in their cellular organisation between different groups of plants. For example, the shoot and root apices of many ferns are characterised by the presence of a large tetrahedral cell, whereas seed plant meristems are multi-layered (Clowes, 1961; Newman, 1965; Philipson, 1990; Popham, 1951). These differences in organisation indicate that the processes of cell division, expansion and differentiation in meristems vary extensively between different groups of plants. From the perspective of plant evo-devo this poses several key questions (Arnoux-Courseaux and Coudert, 2024; Fouracre and Harrison, 2022; Kean-Galeno et al., 2024), including establishing what meristem types are ancestral in major groups of land plants and when the modern diversity of meristem types evolved. Fossils are essential for answering these questions and I now highlight three examples that showcase ways in which fossils can (1) demonstrate trait conservation; (2) aid dating of the advent of key meristematic characteristics; and (3) provide a record of developmental diversity lost to extinction.

### Conservation of meristem structure over 300 million years

**Fig. 2.**
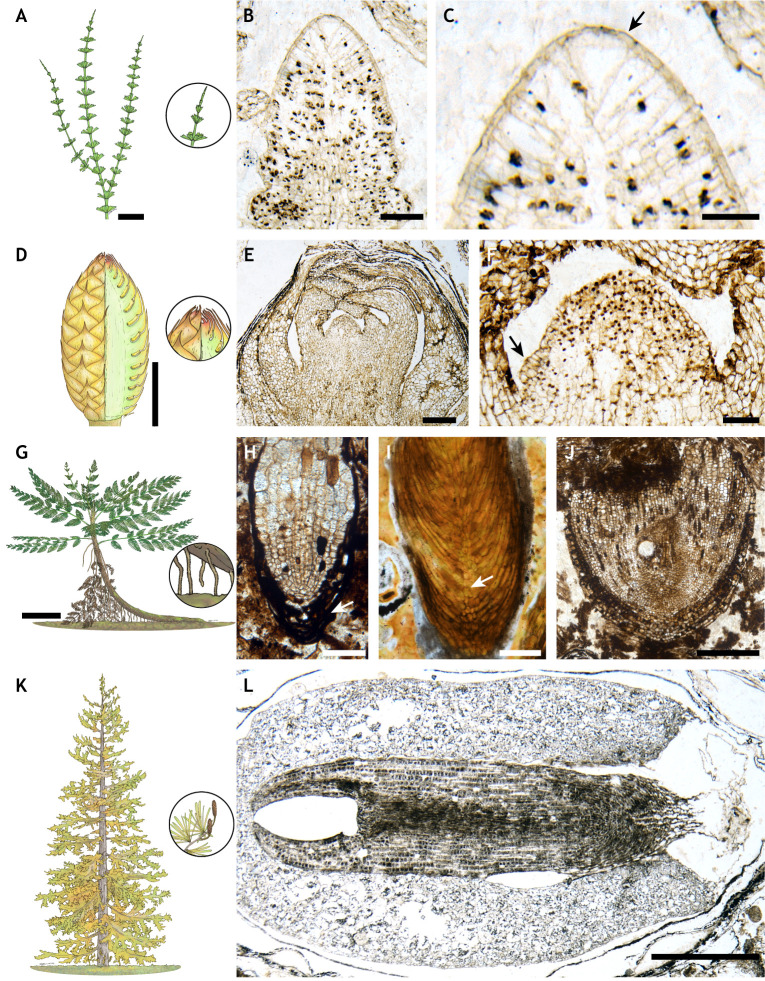
**Exceptionally preserved fossils provide insights into meristem evolution in the geological past.** (A-C) Vegetative shoot apex of *Sphenophyllum* with prominent tetrahedral apical cell, highlighted with arrow in C, similar to those found today in living *Equisetum*. (D-F) Reproductive shoot apex of the male cone of *Lasiostrobus polysacci*. Arrow in F marks the position of a microsporophyll primordia on the flank of the apex. (G,H) Root apex of the early seed plant *Lyginopteris oldhamia* with root cap (highlighted with an arrow in H) covering the tip. (I) Large root cap with distinct columella, highlighted with arrow, of *Araucariorhiza joae*, an extinct conifer. (J) The unique cellular organisation in the broad root apex of *Radix carbonica*. (K,L) *Parasciadopitys aequata*, an extinct conifer with exceptionally preserved seed containing megagametophyte tissue and embryo. Illustrations in A, D, G, K, by Julianne Kiely. Specimen accession codes: (B,C) #673 (636 D bot slide #2), (E,F) #11,489 (2189 E3 Bot slide #90), (L) #26,513 (10160 D1 side #2 slide #10), Division of Paleobotany, Biodiversity Institute, University of Kansas (KUPB), USA. (H) Thin section R646, Manchester Museum, The University of Manchester, UK. (I) Σ 1211 (Museum of the University of Michigan No. 46180)/USNM 274152 Smithsonian National Museum of Natural History, USA. (J) Thin section 81, Oxford University Herbaria, UK. Scale bars: 20 cm (G); 2 cm (A); 1 cm (D); 500 µm (E,J,L); 200 µm (I); 100 µm (B,F,H); 50 µm (C).

Exceptionally preserved shoot apices that include fossilised meristems and the surrounding differentiating tissues from relatives of modern horsetails from the Carboniferous Period (roughly 300 Ma) are marked by the presence of a prominent apical cell. In *Sphenophyllum* (Fig. 2A-C), there is a prominent tetrahedral cell with triangular outer surface and three internal cutting faces (Good and Taylor, 1972), whereas in a related genus, *Calamites*, the cell is five-sided with a square outer surface and four internal cutting faces (Good, 1971). The tetrahedral cell of extinct *Sphenophyllum* and the overall cellular organisation of the broader shoot apex are remarkably similar to those of living *Equisetum* (Bierhorst, 1971; Golub and Wetmore, 1948; Good and Taylor, 1972) (Fig. 2B,C). Parallels have been found in an investigation of the root apex of the extinct fern *Psaronius*. Root apices of *Psaronius* (Ehret and Phillips, 1977) had a varying number of prominent triangular cells when cut in transverse section. The cellular organisation of these root apices is anatomically similar to the root meristems of living ferns today, especially members of the Marattiaceae family (Bower, 1889; Guttenberg, 1966). In both cases, these fossils indicate that the cellular organisation, and by inference the development, of the shoot and root meristems of ferns has been highly conserved for over 300 million years. A similar high level of structural conservation can be seen in the reproductive shoot meristem of seed plants. The cone of *Lasiostrobus polysacci* (Taylor, 1970) (Fig. 2D-F), with its domed reproductive apex and microsporophyll primordia on its flanks (see Fig. 2F for an example of a primordium indicated with arrow on left flank) shares many similarities with those of living conifers (Popham, 1951). The cellular organisation of horsetail shoots, fern roots and conifer cones all provide evidence of high conservation of vegetative and reproductive meristems over the course of 300 million years.

### Charting the origin of major meristematic characteristics

Exceptionally preserved fossils also hold the potential to establish the minimum ages when distinctive features of meristems or developmental processes evolved, an essential step for establishing the temporal evolution of plant development. The evolution of a specific type of shoot branching, termed axillary branching, whereby a new shoot bud develops in the axil where the leaf connects to the shoot, was an important innovation in land plant evolution. The origin of this mode of branching underpins a wealth of architectural form in seed plants and some ferns and fossils demonstrate that it had a minimum point of origin in seed plants at the beginning of the Carboniferous Period of 350 Ma (Galtier and Holmes, 1982). Then, it is found extensively in seed plants, and independently in ferns, by the end of the Carboniferous Period 300 Ma (Brenchley, 1913; Galtier and Holmes, 1982; Galtier and Phillips, 2014; Phillips and Galtier, 2011; Stewart and Rothwell, 1993; Taylor and Stockey, 1976). Fossils, therefore, provide a timescale for the evolution of axillary branching and support the hypothesis that axillary branching evolved independently in ferns and seed plants. Fossil roots demonstrate that the root cap, a key defining feature of the root apices of species today, evolved at least 315 Ma (Fig. 2G,H) (Dennis, 1969; Halket, 1930; Hetherington et al., 2016; Osborn, 1909; Stopes and Watson, 1909), and the distinctive columella of conifer roots extends back at least 210 Ma (Daugherty, 1963) (Fig. 2I). Fossilised roots also preserve evidence for the origins of lateral branching (Hetherington et al., 2020), and even allow us to pinpoint that lateral roots developed in the pericycle before rupturing the endodermis to emerge (Halket, 1932) in the early seed plant *Lyginopteris oldhamia* (Fig. 2G). Such fossils lend support to the hypothesis that lateral roots first developed from the pericycle in seed plants (Singh et al., 2023; Vanneste and Beeckman, 2020; Xiao et al., 2019).

### Extinction of developmental diversity

Finally, one of the most important roles of fossils is to shed light on modes of development that are now entirely extinct or mosaics of characteristics that are not known to co-occur in living species. The cellular organisation of root apices has been extensively studied for over a century (Clowes, 1961; Fujinami et al., 2020; Groot et al., 2004; Heimsch and Seago, 2008; Nägeli and Leitgeb, 1868; Schüepp, 1917; Van Tieghem and Douliot, 1888). Despite this extensive study of living species, the fossil root meristem of *Radix carbonica* (Hetherington et al., 2016) (Fig. 2J) had a cellular organisation that has no living analogue today, including many anticlinal cell divisions within a broad promeristem. This suggests that the mode of root development preserved in this meristem was distinct from any known roots and is now extinct. Similar levels of extinction are also observed in the diversity of fossilised embryos. Fossilised embryos are known from a range of different plant groups, including extinct groups of gymnosperms (Long, 1975; Stockey and Rothwell, 2003), conifers (Mapes et al., 1989; Miller and Brown, 1973), *Araucaria* (Darrow, 1936; Stockey, 1975; Stockey et al., 1992, 1994), and early angiosperms (Friis et al., 2015). The 240 Ma embryo of *Parasciadopitys aequata* (Schwendemann et al., 2010) (Fig. 2K,L), representing an extinct group of conifers called the Voltziales, offers the chance to investigate a key stage in plant development for a group that is now entirely extinct. The embryo preserves a shoot apex with two cotyledons, and a vascular strand extending down to the embryonic root (radicle), with a continuous epidermis running between the cotyledons and the root cap. Interestingly, the embryo includes several characteristics in common with the embryos of living members of the Taxaceae and the Podocarpaceae, but it is distinct from these living groups. Both the fossil root apex and diversity of fossil embryos demonstrate that key developmental differences exist in groups of plants in the past. Collectively, fossils such as these all provide invaluable insights on the evolution of plant development. They help us to constrain the timing of developmental innovations, and demonstrate a juxtaposition of characteristics some of which have been highly conserved over hundreds of millions of years and others that are now entirely extinct.

## Building and testing theories for the evolution of the sporophyte plant body plan

Some of the biggest outstanding questions in the field of plant evo-devo concern the origin of the complex plant body plan, especially the distinctive body plan of the sporophyte (diploid) phase of the life cycle complete with distinct roots, shoots and leaves (Dolan, 2009; Graham et al., 2000; Harrison, 2017; Niklas and Kutschera, 2009; Pires and Dolan, 2012; Rothwell et al., 2014; Schneider et al., 2002). All major groups of living vascular plants possess these characteristics and bryophytes as an outgroup lack them; therefore, living species alone offer limited evidence for predicting how the sporophyte body plan evolved (Fig. 1). Fossils examined in a phylogenetic context offer a vital framework for tackling these questions and provide evidence for combinations of characteristics not found in living species today. Abundant fossil evidence demonstrates that early polysporangiophytes, such as *Aglaophyton majus*, *Horneophyton lignieri* and *Huia gracilis* (Fig. 1), were all composed of branched axes (Kenrick and Crane, 1997), so named to highlight the lack of differentiation between shoots, leaves and roots. The earliest group of plants to develop a body plan with clear delineation between root, shoots and leaves are members of the Drepanophycales order, a group of now extinct lycopsids (Hetherington et al., 2021; Hueber, 1992; Kenrick and Crane, 1997; Matsunaga and Tomescu, 2016, 2017). These plants were globally distributed during the Early Devonian, roughly 400 Ma, and all had similar overall form (Hetherington et al., 2021; Hueber, 1992; Kenrick and Crane, 1997; Matsunaga and Tomescu, 2016, 2017; Xue et al., 2016). Fortunately, *Asteroxylon mackiei* (Fig. 1), a member of this clade was preserved in the Rhynie chert, a fossil site of exceptional preservation (Edwards, 2004; Hetherington et al., 2021; Kerp, 2018; Kidston and Lang, 1920). The combination of its key phylogenetic position, sitting between early extinct vascular plants that lack leaves and roots, and living lycophytes with leaves and roots, as well as its exceptional level of preservation has made *A. mackiei* a model system for investigating the origin of the lycophyte body plan.

**Fig. 3.**
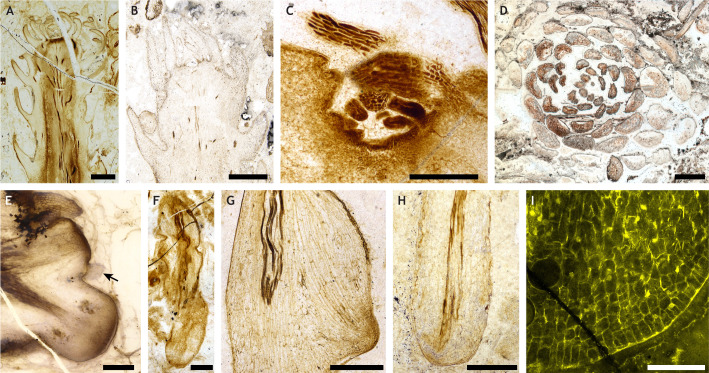
***Asteroxylon mackiei* provides a unique perspective on the evolution of the sporophyte land plant body plan.** (A-I) Diversity of meristems in the extinct 407-million-year-old lycophyte *Asteroxylon mackiei* (see Fig. 1). (A,B) Shoot apical meristems. (C) A lateral shoot meristem. (D) Transverse section through a shoot apex preserving the spiral phyllotaxis of leaves. (E) A branching meristem interpreted as a root-bearing axis. A small-scale leaf, highlighted with an arrow, is preserved on the flank of the upper apex. (F-I) Apices of rooting axes, demonstrating their exogenous origin from a root-bearing axis (G) and the lack of a root cap covering the root tip (H,I). Specimen accession codes: (A) NHMUK V.67866 London Natural History Museum, UK. (B) Bhutta BL29A/40 University of Cardiff, UK. (C,F,I) GLAHM Kid 3080 The Hunterian, University of Glasgow, UK. (D) GLAHM Kid 2554 The Hunterian, University of Glasgow, UK. (E) Pb 2020_02 University of Münster, Germany. (G) NHMUK 16433 London Natural History Museum, UK. (H) NHMUK V.15642 London Natural History Museum, UK. Scale bars: 2 mm (A); 1 mm (D); 500 µm (C,E-H); 200 µm (B); 100 µm (I).

Based on 100 years of study (Edwards, 1994, 2004; Hetherington et al., 2021; Kerp, 2018; Kerp et al., 2013; Kidston and Lang, 1920, 1921; Lyon, 1964; Turner et al., 2023), *A. mackiei* likely represents the fossil species for which we have the most available data for its fossilised meristems (Fig. 3). Based on the examination of these meristems, it has been possible to make predictions about development in *A. mackiei* and more widely for the evolution of development in the lycophyte lineage. The shoot apical meristem of *A. mackiei* was similar in structure to those found in living members of the Lycopodiaceae (Bierhorst, 1971; Gola and Banasiak, 2016; Gola and Jernstedt, 2011; Guttenberg, 1966; Imaichi, 2008) (Fig. 3A,B). In addition, microphyll leaves developed on the flanks of the meristems in two main phyllotactic types – either whorls or spirals. As in living members of the Lycopodiaceae, phyllotaxis was highly variable in *A. mackiei* and non-Fibonacci spiral arrangements were common compared with their low frequency in the vast majority of other groups of vascular plants (Gola, 1996; Gola and Banasiak, 2016; Turner et al., 2023; Yin and Meicenheimer, 2017) (Fig. 3D). Broadly, the shoot development of *A. mackiei* is similar to that seen in living lycophytes, although differences do exist, especially in the development of reproductive structures. However, the development of the rooting system of *A. mackiei* was significantly different to that of living species. Roots in living members of the Lycopodiaceae typically develop endogenously, whereby the root meristem develops from internal cell layers and actively grows through older tissue layers to erupt, and develop from a root meristem with root cap (Hetherington and Dolan, 2017). In contrast, the rooting system of the *A. mackiei* and other members of the Drepanophycales, such as *Sengelia radicans* (Matsunaga and Tomescu, 2016, 2017), were composed of two distinct parts: rooting-bearing axes and rooting axes. The exceptionally preserved anatomy of *A. mackiei* provided the anatomical evidence to demonstrate that neither root-bearing axes nor rooting axes developed endogenously or developed from a root meristem with root cap (Fig. 3E-I) (Hetherington and Dolan, 2018; Hetherington et al., 2021).

In *A. mackiei*, rooting systems are first distinguishable at branch points where the smaller branch formed at a dichotomous branching point lacks the characteristic shoot meristem with dense cluster of leaves (Fig. 3A-C) and instead develops a meristem with sparse smaller scale leaves (Fig. 3E). In overall form, these axes, termed root-bearing axes, showed pronounced downward growth and developed only a few characteristics of the leafy shoot, such as a cuticle, occasional scale leaves and stomata (Hetherington et al., 2021). Attached to these root-bearing axes, again at dichotomous branch points, were smaller highly branched axes that resemble the roots of living lycophytes. However, careful examination of their meristems at different stages of development demonstrate that they are distinct from roots in living lycophytes (Fig. 3F-I). First, their origin was exogenous rather than the endogenous origin of many living lycophyte roots (Fig. 3G), and, most crucially, they developed from a root meristem that lacked a root cap (Fig. 3H,I) (Hetherington and Dolan, 2018; Hetherington et al., 2021). The lack of the root cap means they cannot be considered true roots and instead are interpreted as a transitional rooting axis that developed many – but not all – of the features that characterise the development of true roots. This finding provided compelling evidence that roots with root caps had two independent origins in vascular plants, once in lycophytes and separately in euphyllophytes.

In summary, today, no comparable rooting systems to that preserved in *A. mackiei* and other members of the extinct Drepanophycales exist, but, crucially, the fossils of these species preserve a combination of characteristics observed in roots and shoots of living species. Overall, *A. mackiei* provides evidence for a stepwise origin of the characteristics that distinguish roots and leafy shoots in living species and demonstrates that changes in axis type occur at branching points. This suggests a key role of unequal branching events for the development of complex body plans in lycophytes. Fossils such as *A. mackiei*, therefore, shape our understanding of the evolution of the sporophyte body plan in land plants and help frame hypotheses that, in turn, can be tested in living species (Fujinami et al., 2021).

## Perspectives

Fossils provide unique insights into many of the biggest questions in plant evo-devo, such as the origin of plant body plans or the developmental changes that underpin the evolution of roots, leaves, wood and seeds. The advances that can be gained by their inclusion far outstrip the inherent limitations of the fossil record, whether owing to the challenges of sample size or our inability to extract ancient DNA. Fossils, such as those highlighted here, demonstrate the level of developmental information that can be preserved within sites of exceptional preservation and the conclusions that can be drawn when these fossils are integrated into a broader phylogenetic context alongside the diversity of living species today. Finally, although sites of exceptional preservation are inherently rare in the fossil record and many have been discovered and examined for over 100 years, continued research on these sites remains essential and promises to provide hugely valuable insights in the future. At a time when the number of sequenced genomes and genetically tractable plant species are increasing and allowing the investigation of major questions across different groups of plants, the integration of fossils into studies in plant evo-devo is more important than ever before. Leveraging this new suite of genetically tracible model plant species (Ariyarathne et al., 2024; Frangedakis et al., 2021; Muthukumar et al., 2013; Plackett et al., 2014) will allow us to functionally test predictions about the evolution of the land plant body plan in unprecedented detail. Studies concerning the origin, evolution and development of leaves in vascular plants, where there is a strong history for building hypotheses using an integrated framework of fossils and living species (Boyce, 2005, 2008, 2010; Boyce and Knoll, 2002; Floyd and Bowman, 2006, 2010; Harrison and Morris, 2018; Harrison et al., 2005; Kenrick, 2002; Kenrick and Crane, 1997; Rothwell et al., 2014; Sanders et al., 2007; Tomescu, 2009; Tomescu and Whitewoods, 2024; Turner et al., 2023; Vasco et al., 2013, 2016; Zimmerman, 1952), will provide an ideal test case for the future and are primed to functionally test predictions with genetically tractable ferns and lycophytes (Ariyarathne et al., 2024; Muthukumar et al., 2013; Plackett et al., 2014).
